# Global or local modeling for XGBoost in geospatial studies upon simulated data and German COVID-19 infection forecasting

**DOI:** 10.1038/s41598-025-92995-6

**Published:** 2025-03-14

**Authors:** Ximeng Cheng, Jackie Ma

**Affiliations:** Department of Artificial Intelligence, Fraunhofer Heinrich Hertz Institute, 10587 Berlin, Germany

**Keywords:** Machine learning, Time-series forecasting, Spatial heterogeneity, Spatial partitioning, Spatial variation, Ecological epidemiology, Sustainability

## Abstract

Methods from artificial intelligence (AI) and, in particular, machine learning and deep learning, have advanced rapidly in recent years and have been applied to multiple fields including geospatial analysis. Due to the spatial heterogeneity and the fact that conventional methods can not mine large data, geospatial studies typically model homogeneous regions locally within the entire study area. However, AI models can process large amounts of data, and, theoretically, the more diverse the train data, the more robust a well-trained model will be. In this paper, we study a typical machine learning method XGBoost, with the question: Is it better to build a single global or multiple local models for XGBoost in geospatial studies? To compare the global and local modeling, XGBoost is first studied on simulated data and then also studied to forecast daily infection cases of COVID-19 in Germany. The results indicate that if the data under different relationships between independent and dependent variables are balanced and the corresponding value ranges are similar, i.e., low spatial variation, global modeling of XGBoost is better for most cases; otherwise, local modeling of XGBoost is more stable and better, especially for the secondary data. Besides, local modeling has the potential of using parallel computing because each sub-model is trained independently, but the spatial partition of local modeling requires extra attention and can affect results.

## Introduction

**Fig. 1:**
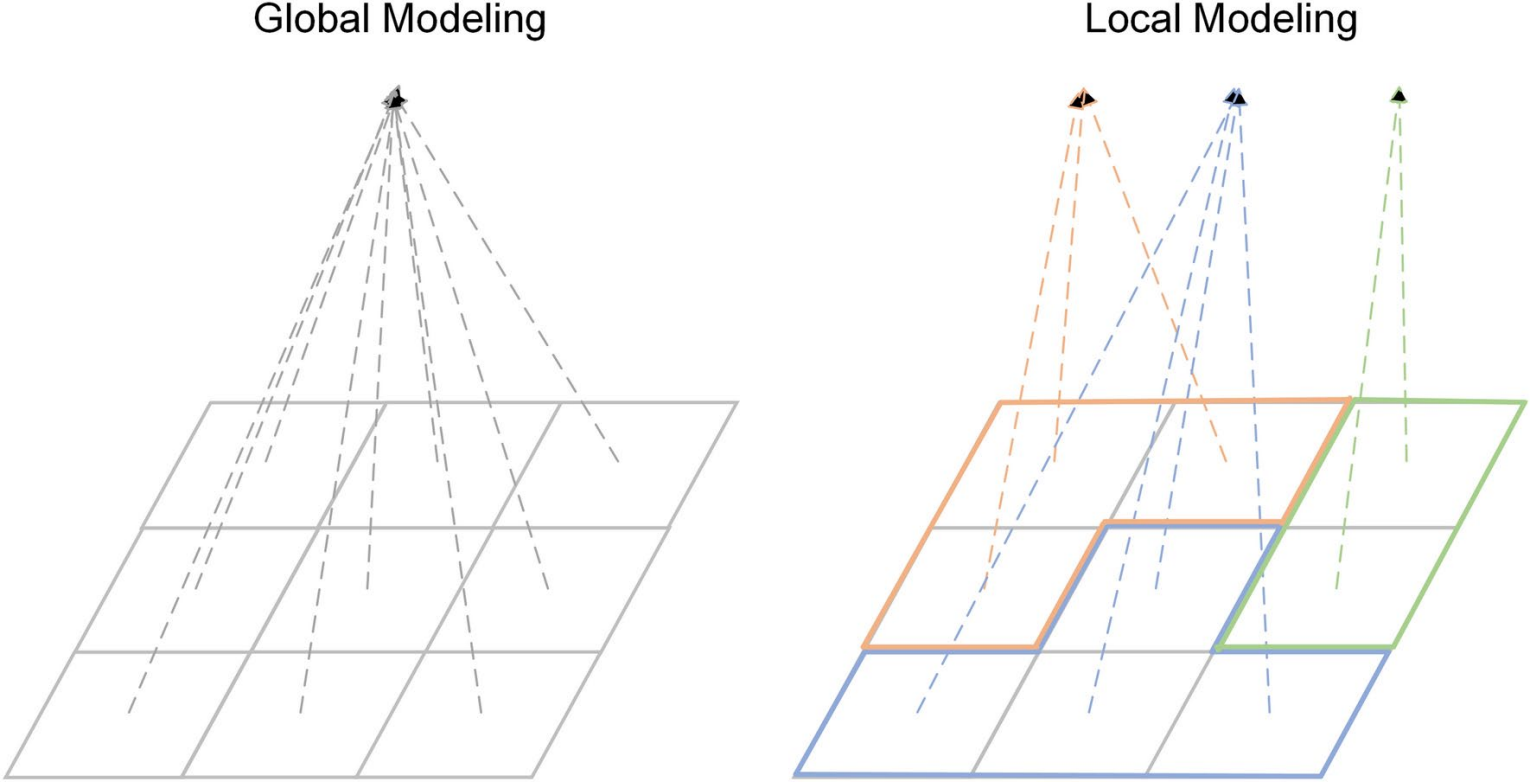
Schematic diagram of global- and local modeling.

Due to the rapid development of information and communication technologies, huge data recording spatio-temporal information can be collected and can be used for research, which also promotes the wide application of artificial intelligence (AI) methods in geospatial studies^1,2^. AI techniques represented by machine learning and deep learning methods have powerful capabilities in data mining^3^. They can achieve good performance on multiple tasks that also benefit the development of specific fields, and thus sub-disciplines such as geospatial artificial intelligence (GeoAI) have emerged^4^. Geospatial research has some inherent spatial properties including spatial heterogeneity, which means that the investigated relationships may vary significantly with the study area. Different from the general statistical studies, it is impossible to detect an average place on Earth^5^. Therefore, studies that applied conventional geospatial methods often adopt local modeling, that is, separate modeling for specific areas with significantly different environmental characteristics. But for AI methods such as deep learning, its complex neural network structure and a large number of parameters may be able to solve the problems spatial heterogeneity brings. Besides, diverse train data can also improve the robustness of the AI model. Therefore methods from AI are also frequently applied for global modeling (i.e., training one model based on the data in the entire study area) in geospatial studies. In Fig. 1 we visualize the two different modeling approaches.

However, current AI application studies pay more attention to designing new methods, adding new train data, and exploring the interpretability of models^6,7^, but less discuss the comparison of the differences mentioned above of global- and local modeling. In computer science, it is sometimes easier to decompose a complex task into multiple sub-modules^8,9^ that are being individually solved. Each module can be responsible for achieving a specific simple sub-task, which can be regarded as local modeling from the functional perspective. In terms of software defect prediction, several studies compared the performance of the two modeling ways (i.e., local- and global) from the data perspective, but the conclusions are, as expected, inconsistent. For instance, the study in^10^ shows improvement in the local modeling, whereas in^11^ both approaches are comparable. In^12^ the authors have shown advantages for both approaches. Concerning geoscience, there are also hydrological studies comparing the two modeling ways of AI methods. Fang et al.^13^ found that the global- performs better than the local modeling for deep learning models in predicting soil moisture and streamflow. Morgenstern et al.^14^ compared the local- and regional (the train data are the combinations of several catchments) modeling by using the long short-term memory (LSTM) networks^15^. Kratzert et al.^16^ found that the global modeling of machine learning methods performs better than the local- and regional hydrological benchmark models. Clark et al.^17^ summarized that local- and global modeling ways both have drawbacks and benefits by trying multiple methods in time-series forecasting tasks. Given the current relatively scarce research status of the two modeling ways of AI methods in geospatial studies, we compare both approaches based on simulated data and also for the task of COVID-19 infection forecasting in this work.

The eXtreme Gradient Boosting (XGBoost) algorithm^18^ is a popular machine learning method that has been applied in multiple fields, including COVID-19 studies^19^. The XGBoost is a kind of ensemble learning method, which trains multiple submodels (e.g., Decision Tree) and summarizes all the outputs to achieve one task. Existing studies have shown that in many tasks, XGBoost performs comparably to deep learning models^20,21^, and also has the advantage of interpretability. In addition, based on the consideration of fairly comparing global- and local modeling approaches, we select the XGBoost algorithm which has a shorter training time and fewer model parameters, instead of deep learning models to conduct comparative studies of simulation data and the case of German daily COVID-19 infection forecasting. The experiments based on simulated data are controllable and they can analyze the impact of a single factor (e.g., sample size) in two modeling ways, while experiments based on real data (i.e., German COVID-19 infections) provide an opportunity to analyze the spatio-temporal factors in actual geospatial studies. It should be noted that this study is not to pursue better forecasting performance in general, so for the global modeling we do not add extra data (e.g., traffic flow between cities) nor do we use more elaborated methods such as graph convolutional neural networks^22^ to compare with local modeling in the real case.

## Results

### Global- and local modeling for XGBoost on simulated data

This experiment generates data X and Y based on the linear function, the power function, and the trigonometric function (i.e., Eqs. 1, 2, and 3), and compares the performance between global- and local modeling ways of XGBoost from three aspects: data distribution, sample size, and value range. The details of experimental design and method implementation are provided in the “Methods” section. It is worth mentioning that all the results shown in this section are the average values of 100 times independent simulation. For each experiment, two types of data are generated under two of the three equations, and they are combined as the entire data set for the comparison between global- and local modeling approaches. In addition to comparing the model performance on the entire data, the prediction performance on the two types of data subsets (e.g., *G*1 and *G*2 displayed in Table 1) has also been investigated for a detailed analysis.

**Table 1:** Average RMSE for the simulated data under two distributions.

Distribution	Equation	Global	Local	G1	L1	G2	L2
Normal	Linear+Power	33.362	33.305	34.457	33.339	32.029	33.265
	Linear+Trigonometric	36.305	33.495	39.439	33.408	32.520	33.575
	Power+Trigonometric	32.551	33.533	32.646	33.541	32.449	33.518
Pareto	Linear+Power	29.868	31.239	30.437	31.579	29.283	30.889
	Linear+Trigonometric	30.214	31.343	30.261	31.258	30.159	31.419
	Power+Trigonometric	30.270	31.364	30.151	31.091	30.381	31.630

*G*1 and *G*2 denote the performance of global modeling on simulated data subsets under the first and second equations. The same applies to *L*1 and *L*2 for the local modeling.

Table 1 displays the average root mean squared error (RMSE) of XGBoost using global- and local modeling ways on simulated data by different probability densities (i.e., Normal distribution and Pareto distribution) of resampling. It reflects that the results of both global- and local modeling ways based on Pareto distribution data are better than the ones based on Normal distribution data. This is due to the different characteristics of the two distributions. The data under the Normal distribution are concentrated in the middle, while data under the Pareto distribution are mostly with low values. Therefore, when the value range is the same, it is reasonable that the absolute values of the statistical indexes (e.g., average RMSE) of Parteo distribution data are smaller than that of Normal distribution. Comparing global- and local modeling ways, local modeling obtains better results for the two equation combinations based on Normal distribution data, and the result of the other combination is also similar. The global modeling achieves better results in all three equation combinations based on Pareto distribution data. Specifically for the performance on each sub-data, local modeling only beats global modeling under the linear function based on Normal distribution data, and all other cases are worse than global modeling. In summary, when the data are balanced and the value range is consistent, the difference in data distribution will affect the absolute value of model performance indexes; for the two modeling ways, global modeling works better in more cases, while local modeling works better in specific cases of this experiment (e.g., under the linear function based on Normal distribution data). Considering that data with Normal distribution appears frequently in research and its corresponding statistical indicators (e.g., average value) can effectively reflect the overall information of the data, only Normal distribution will be used to resample data in subsequent experiments.

**Fig. 2:**
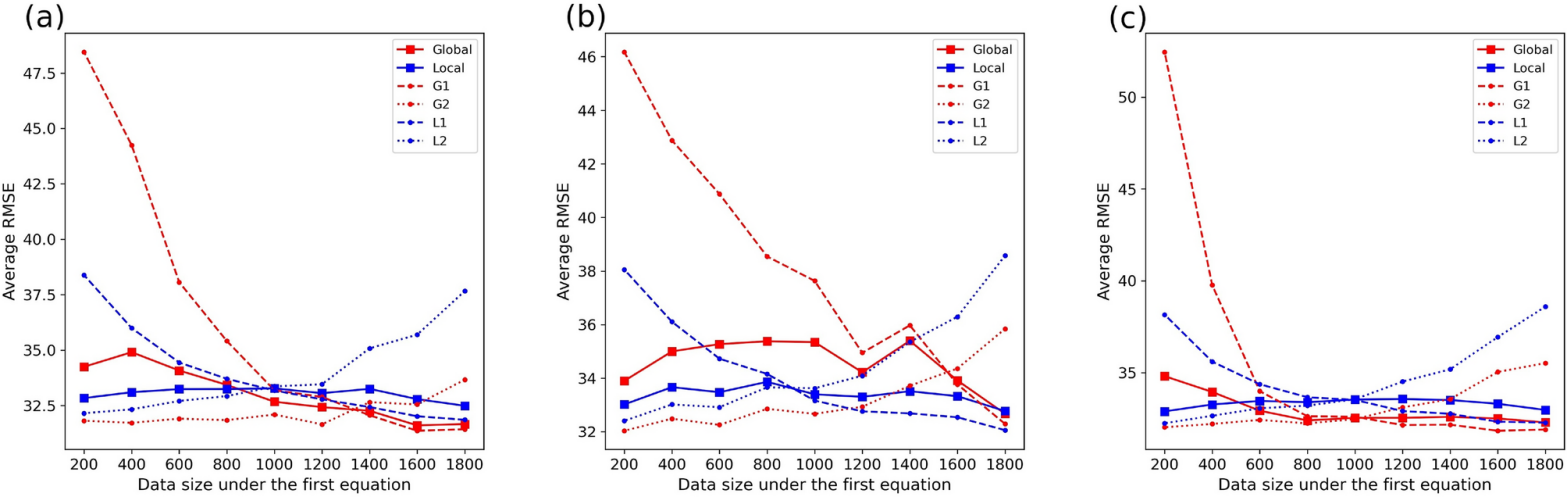
Global- and local modeling on different sizes of simulated data (**a** Data generation under linear and power equations; **b** Data generation under linear and trigonometric equations; **c** Data generation under power and trigonometric equations). The total size of train data is equal to 2000. The x-axis shows the data size under the first equation for each combination. *G*1 and *G*2 denote the performance of global modeling on simulated data subsets under the first and second equations. The same applies to *L*1 and *L*2 for the local modeling.

To compare global- and local modeling approaches based on train data with different sizes, we control the total data size under two equations to 2000 and set the data size under the first equation to 200, 400, 600, 800, 1000, 1200, 1400, 1600, and 1800. The model performance of the two modeling ways can be compared under different conditions with balanced data (e.g., the data size for both subsets is equal to 1000) and unbalanced data (e.g., the data sizes for two subsets are 200 and 1800). Figure 2 displays the variation in model performance based on different sizes of train data under three combinations of equations (i.e., Linear+Power, Linear+Trigonometric, and Power+Trigonometric). It reflects that for any sub-data, the model performance for both modeling ways will improve with the increase of corresponding data size, and vice versa. This is reasonable because rich data will provide much information and make the training of AI models sufficient. Comparing global- and local modeling ways, the model performance of the two is similar when the case is under the combination of linear and power equations. Local modeling way is better under the combination of linear and trigonometric equations, while global modeling way is better for most cases under the combination of power and trigonometric equations. This reflects that there is no eternal winner of the two modeling ways in cases with different data sizes. However, it can also be found that the performance of global modeling is more sensitive to data with small sizes than the one of local modeling. This is because the training by the global modeling way is to pursue the optimality for the loss function of the entire data, so it may focus more on predicting the main data and ignore the secondary samples under another equation. The local modeling way achieves a stable performance on data with small sizes because it trains sub-models independently for data under different equations and pursues local optimum.

To compare the predicting performance of the global- and local modeling ways based on the data under different value ranges, we take the combination of linear and power equations as an example to conduct experiments. The value range of the variable *Y* in one of the equations is controlled to 0–1000 without noise, and the weight *W* in another equation is changed to make the value range of *Y* without noise to 0–1000, 0–2000, 0–4000, 0–10,000, 0–20,000, 0–40,000, 0–100,000, and 0–200,000. Table 2 shows the average RMSE results of the two modeling ways. The results reflect that as the maximum *Y* increases, the corresponding RMSE will also increase for both global- and local modeling ways, which is in line with common sense. To further observe the model performance on sub-data, we find that the increase in RMSE for the entire dataset comes more from the data that changed the value range, while the performance on the other unchanged data is stable for both global- and local modeling ways. The only exception in this experiment is that when the maximum *Y* of data under the Power equation is extremely large, the global modeling performance on unchanged data under the linear equation will also deteriorate. Comparing the two modeling ways, they achieve similar performance when the value range changes little, while the local modeling way performs better when the value range changes greatly (i.e., maximum *Y* is larger than 40,000).

**Table 2:** Average RMSE for the simulated data under different value ranges.

Change	Max Y without noise	Global	Local	G1	L1	G2	L2
Linear	1000	32.681	33.414	33.233	33.366	32.076	33.455
	2000	33.656	33.830	35.234	34.242	31.928	33.406
	4000	35.039	34.671	37.839	35.863	31.907	33.429
	10,000	36.020	35.640	39.932	37.859	31.587	33.262
	20,000	37.996	38.666	43.646	43.195	31.336	33.515
	40,000	48.876	47.500	61.840	58.262	30.804	33.385
	100,000	101.042	90.513	138.941	123.588	31.854	33.141
	200,000	185.717	167.474	260.740	234.468	30.985	33.313
Power	1000	32.422	33.261	33.117	33.344	31.681	33.173
	2000	32.440	33.739	32.048	33.424	32.820	34.044
	4000	33.241	34.335	31.930	33.307	34.493	35.326
	10,000	35.145	35.786	31.662	33.226	38.303	38.168
	20,000	38.762	38.703	31.534	33.412	44.827	43.338
	40,000	49.652	48.131	32.550	33.520	62.168	59.202
	100,000	96.579	90.528	38.677	33.399	130.866	123.556
	200,000	185.663	170.368	55.178	33.298	256.426	238.600

*G*1 and *G*2 denote the performance of global modeling on simulated data subsets under the first and second equations. The same applies to *L*1 and *L*2 for the local modeling

In geospatial studies, to make accurate statistical analysis and better training models based on data under non-Normal distributions, researchers will transform the raw data (e.g., log transformation) to make its distribution more consistent with the Normal distribution. Therefore, we also transform the *Y* in generated data into logarithmic forms to investigate the impact of the value range on the two modeling ways further. Based on the existing experiments whose results are displayed in Table 2, we utilize the nature log ln to transform the *Y* of the data generated under the linear equation for both train and test data. After model training, the predicted *Y* needs to be transformed back using exponential transformation to make the absolute RMSE values comparable with the ones in previous experiments. Table 3 shows the average RMSE of global- and local modeling ways based on data using log transformation. The results are similar to the ones displayed in Table 2. The only difference point is that after the log transformation, the model performance becomes worse when the maximum *Y* is large. It reflects that the simple value transformation cannot improve the model performance of either modeling approach.

**Table 3:** Average RMSE for the simulated data under different value ranges with log() transformation.

Change	Max Y without noise	Max log(Y)	Global	Local	G1	L1	G2	L2
Linear	1000	6.908	32.601	33.215	32.355	33.132	32.764	33.290
	2000	7.601	33.245	33.682	33.693	34.057	32.727	33.293
	4000	8.294	34.685	33.900	36.266	34.524	32.918	33.255
	10,000	9.210	39.367	35.219	45.030	37.171	32.708	33.144
	20,000	9.903	52.967	38.928	67.235	43.722	32.952	33.436
	40,000	10.597	91.498	50.596	125.147	63.326	32.780	33.264
	100,000	11.513	219.612	101.889	308.853	140.218	32.560	33.090
	200,000	12.206	438.299	193.396	618.954	271.442	33.059	33.345

*G*1 and *G*2 denote the performance of global modeling on simulated data subsets under the first and second equations. The same applies to *L*1 and *L*2 for the local modeling

To summarize the results of global- and local modeling ways of XGBoost on simulated data, the two ways have specific advantages and the modeling approach selection should according to the actual situation in geospatial studies.

- Global modeling performs better in most cases when the data under different relationships are balanced and the corresponding value range is similar, i.e., the degree of spatial variation is low;
- Local modeling can achieve more stable and better performance when the data under different relationships are unbalanced and the corresponding value ranges are varied greatly, i.e., the degree of spatial variation is high, especially for the secondary data.

It shows that it is necessary to compare global- and local modeling ways of applying XGBoost to geospatial analysis tasks (e.g., COVID-19 infection forecasting), under possible unbalanced train samples and various value ranges.

### Global- and local modeling for XGBoost on German COVID-19 infection forecasting

The introduction of data and experimental details are provided in the “Methods” section. Based on the optimized parameters, we train several XGBoost models to forecast COVID-19 infections in global- and local modeling ways. Four evaluation indicators including mean absolute error (MAE), mean squared error (MSE), RMSE, and Cosine similarity (Cosine) are used to compare model performance. Table 4 displays the average value of each indicator in 400 regions. The global- and local modeling ways of XGBoost can achieve similar performance. The global modeling performs better in indicators MAE, RMSE, and Cosine, but worse in MSE.

**Table 4:** Comparison of forecasting performance between global- and local modeling ways.

Modeling	MAE	MSE	RMSE	Cosine
Global	**26.381**	3070.200	**31.452**	**0.750**
Local	27.291	**2953.607**	32.430	0.745

Better results are in bold.

**Fig. 3:**
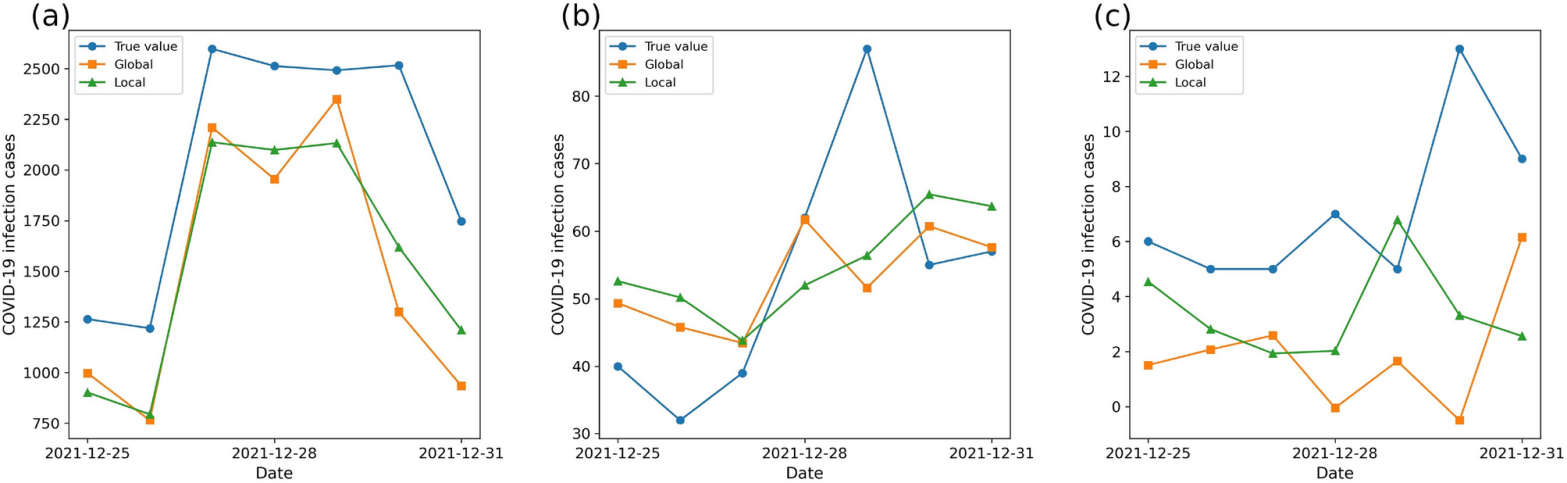
Daily COVID-19 infection forecasting results of global- and local modeling in three regions (**a** Berlin; **b** Ansbach district; **c** Wittmund). These three regions have the maximum, median, and minimum values of the true average infected cases in 7 days.

In terms of the temporal dimension, Fig. 3 displays the true values and the forecasting results of two modeling ways in three regions, which are Berlin, Ansbach district, and Wittmund with the maximum, median, and minimum true infected cases in 400 regions. For the changing trend of time series, the larger the infected numbers in a region, the more similar the predicted time series is to the true value. For forecasting results in Berlin (Fig. 3a), both two models predict a trend in which the infected cases will first increase, then maintain, and finally decrease in 7 days, but the time-series patterns in Wittmud (Fig. 3c) of two models’ results and true values are irregular. This may be due to that the objective of parameter optimization and model training in this experiment is to reduce the RMSE between true values and predicted results (i.e., focusing more on the difference of absolute infected numbers), thus ignoring the difference in the changing trend of time series. Besides, the time-series results of global- and local modeling ways have no obvious difference in temporal patterns in these three example regions.

**Fig. 4:**
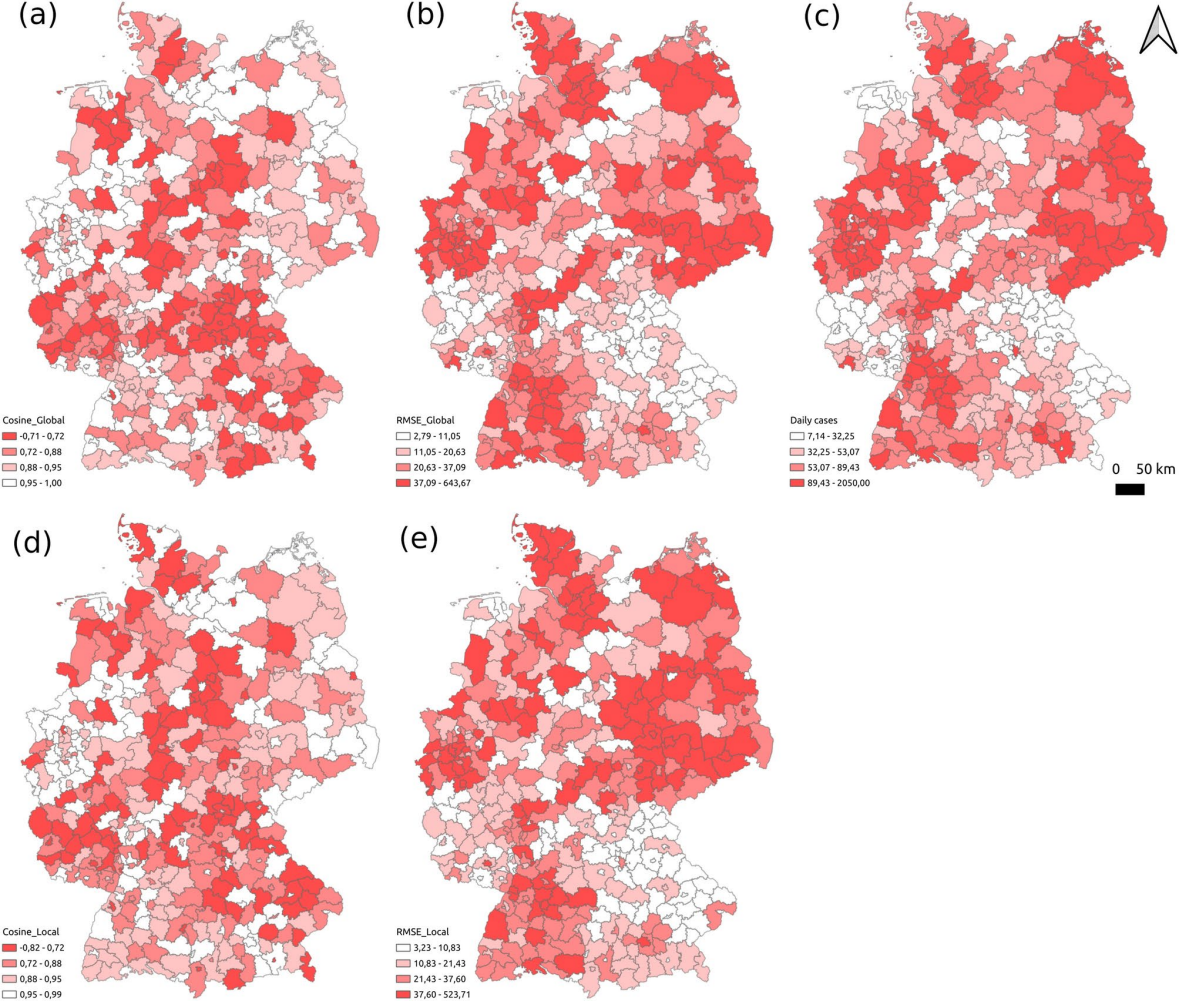
Spatial distributions of the forecasting performance of the global- and local modeling ways (**a** the Cosine similarity between predicted and true values by global modeling; **b** the RMSE between predicted and true values by global modeling; **c** the average daily COVID-19 infected cases in test data; **d** the Cosine similarity between predicted and true values by local modeling; **e** the RMSE between predicted and true values by local modeling). For figures (**a**), (**b**), (**d**), and (**e**), the darker the color, the worse the forecasting of the corresponding region.

In terms of the spatial dimension, we draw the spatial distributions of two indicators Cosine and RMSE that evaluated the absolute error and relative error, respectively. Figure 4 displays the results of two indicators by using the global modeling and the local modeling ways, and the true average COVID-19 infected cases. Based on Fig. 4, we find that the spatial distribution of true values is similar to the ones of RMSE but opposite to that of Cosine [e.g., east Germany is in dark color in figures (b), (c), (e), but in light color in figures (a), (d)]. It indicates that the larger the true infected cases in a region, the higher the absolute errors and the lower the relative errors in forecasting, which is reasonable and consistent with the temporal patterns in Fig. 3. Comparing the forecasting results between global- and local modeling, they have similar spatial distributions in both indicators RMSE and Cosine.

Based on the comparison results in overall performance, temporal dimension, and spatial dimension, the global- and local modeling ways of XGBoost have no obvious difference in the task of German COVID-19 infection forecasting, but this doesn’t mean that comparing the two modeling ways is meaningless. Table 9 provides information on train data size and the value range of samples for each model. Compared with the simulation experiment, the train data difference between the two modeling ways is not significant, especially in terms of the value range, which will cause the performance of local- and global modeling ways of XGBoost are similar in COVID-19 infection forecasting. Besides, spatial partitioning can also affect the performance of local modeling. The current experiments of local modeling are just based on the German Nomenclature of Territorial Units for Statistics (NUTS) (https://ec.europa.eu/eurostat/web/nuts/overview) 1 boundary with an artificial assumption that the inherent patterns of COVID-19 infection are homogeneous within regions but heterogeneous between areas.

## Discussion

In terms of efficiency, we record the consumed time in parameter tuning and model training for both ways of XGBoost modeling (i.e., global- and local modeling) (Table 5). For local modeling, we independently train 16 models according to the state of Germany. Table 5 shows that the global modeling consumes more time in tuning parameters (i.e., 16503.545s) than the local modeling way, but it has the shortest consumed time (i.e., 76.441s) to train a model based on the obtained parameters. The global modeling way has to process larger amounts of train data (i.e., data of the entire study area) than the data of local modeling (i.e., part data of sub-area) for model tuning, but it only needs to train a single model. From the perspective of practical applications, the advantage of global modeling is that it doesn’t divide the study area into several partitions and re-organize the data for model training. Global modeling can quickly obtain results based on general parameters, which facilitates early exploration of the task. In terms of the local modeling, the multiple local models are trained independently and have no data sharing and crossover, which reflects the potential to save time in tuning parameters and training models by using techniques such as parallel computing. This makes the consumed time of local modeling not the sum but the maximum of all sub-models. For instance, in Table 5, the time for tuning parameters and modeling training of the local models will be 3602.481s and 25.380s by parallel computing. It can surpass the global modeling way and is especially suitable for time-limited tasks.

**Table 5:** Comparison of time consumption between global- and local modeling ways.

Modeling		Tuning parameters time (s)	Model training time (s)
Global		16,503.545	**76.441**
Local	1	1617.999	5.124
	2	3602.481	25.380
	3	82.377	0.193
	4	578.380	6.988
	5	114.869	0.270
	6	94.861	0.573
	7	641.196	7.212
	8	277.128	1.423
	9	1542.386	5.926
	10	1851.949	11.737
	11	1134.130	15.223
	12	280.670	1.164
	13	421.114	2.463
	14	474.865	1.488
	15	421.902	3.639
	16	654.389	1.557
	total	**13,790.697**	90.361

Less time are in bold.

To further compare global- and local modeling ways of the XGBoost method in the task of COVID-19 infection forecasting, we use Moran’s *I*^23,24^ to analyze the spatial efforts of the evaluation indicators (i.e., spatial distributions of RMSE and Cosine). Moran’s *I* can measure the spatial auto-correlation of targeted data and has been applied in multiple fields including the studies of COVID-19^25^. The value range of global Moran’s *I* is from -1 to 1, a positive value indicates the clustered spatial pattern, and a negative value indicates the dispersed spatial pattern. We calculate global Moran’s *I* indexes of true daily infected cases and the RMSE and Cosine indicators of the global modeling way and the local modeling way. The implementation of the calculation is based on the Spatial Analysis Toolbox (https://github.com/parmendel/spatialanalysistoolbox), a Python plugins repository for QGIS software. Table 6 displays the results of global Moran’s *I*.

**Table 6:** Global Moran’s *I* of spatial distributions of true infected cases, RMSE, and Cosine.

Data		Moran’s *I*	P-value	Z-score	Confidence
Infected cases		0.095	<0.01	>2.58	99%
Global	RMSE	0.140	<0.01	>2.58	99%
	Cosine	0.089	<0.01	>2.58	99%
Local	RMSE	0.188	<0.01	>2.58	99%
	Cosine	0.094	<0.01	>2.58	99%

In Table 6, the global Moran’s *I* results of all the data are positive with high confidence, which indicates that all the spatial distributions have a significant clustered pattern. The indexes of true COVID-19-infected cases are positive, which means regions with high or low infected cases have neighboring areas with similar high or low infected cases. This is reasonable because COVID-19 infections can spread through space, so the severity of the epidemic in adjacent areas tends to be similar. In terms of the evaluation indicators of models, the corresponding spatial distributions should be random (i.e., global Moran’s *I* tends to be zero or does not have significant spatial patterns) if the trained model fully considers spatial features. In this study, we focus more on fairly comparing global- and local modeling ways of XGBoost, therefore the spatial data such as traffic flows and railway networks are not included in the train data. Compared with the global Moran’s *I* of the infected cases (i.e., 0.095), the two modeling ways seem to intensify the spatial clustered pattern, while the indexes of global modeling are slightly lower than the ones of local modeling. This is because although there is no explicit spatial data for model training, the global modeling considers the data of the entire study area, while the local modeling ignores the data outside its corresponding sub-area. Besides, even though all data have spatial clustered patterns reflected by Moran’s *I*, the value is relatively low, which reflects a slight spatial variation. In addition, the data volume for each county is the same in this study. Those reasons make that the difference between global- and local modeling ways of XGBoost in the task of German COVID-19 infection forecasting is not obvious.

**Fig. 5:**
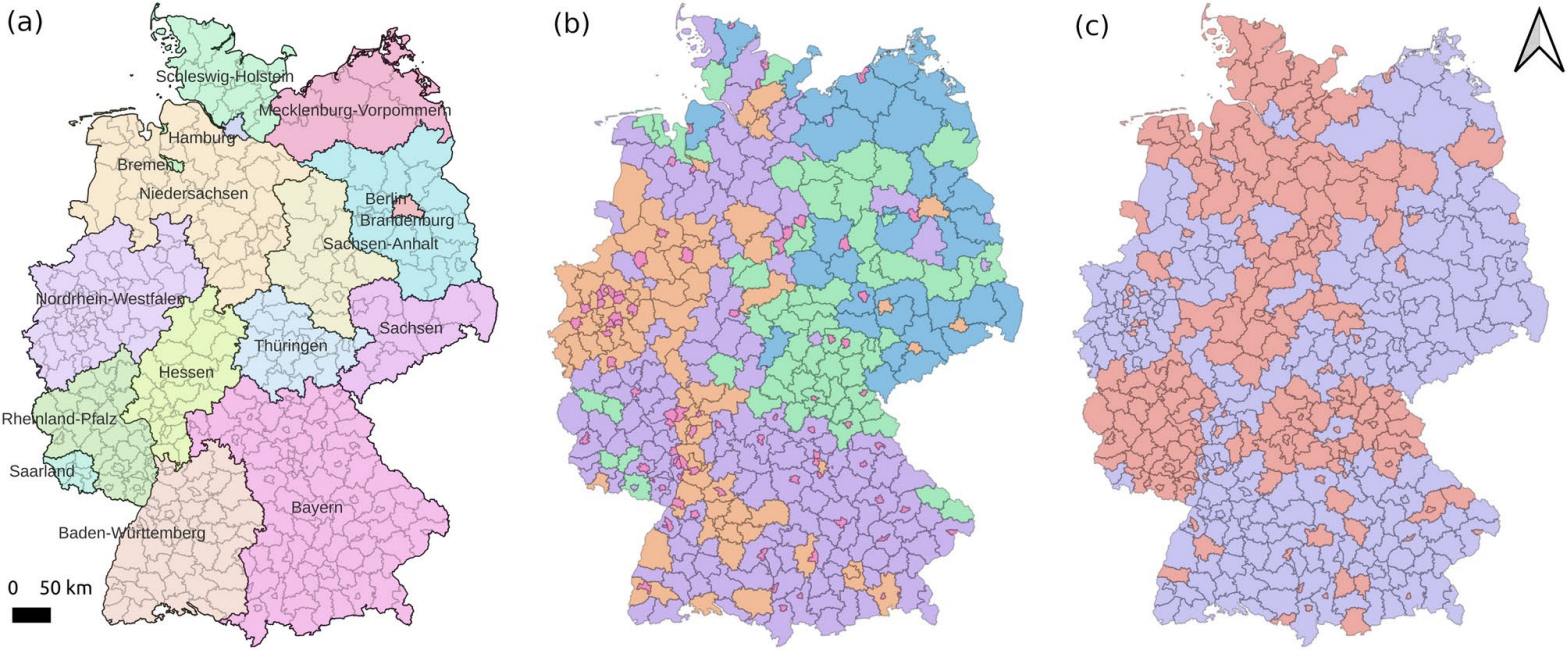
Three spatial partitions of regions in NUTS 3 level, Germany (**a** based on the administrative division in NUTS 1 level, Germany; **b** based on the clustering of static environmental features; **c** based on the clustering of COVID-19 infection case time-series in train data). Data of regions with the same color are used to train one local model.

To further investigate the impacts of spatial partitions on local modeling, besides the partition based on NUTS 1 (denoted as Local_1), we select two more spatial partitions for local modeling, which are created based on multiple static features of 400 regions [e.g., Gross domestic product (GDP), population, median age, and volumes of private vehicles] (denoted as Local_2), and the time series of COVID-19 infection cases during the period of train data (denoted as Local_3). The reason for choosing these additional two is to characterize the spatial heterogeneity of COVID-19 infection patterns based on environmental attributes and observed infection time series. Except for the first one that can be directly determined, the latter two spatial partitions are generated using the spectral clustering method^26^, and the optimal cluster number of each clustering is determined by the Silhouette Coefficient^27^. The algorithm is implemented by the scikit-learn library^28^. Figure 5 shows the spatial partitions of three local modeling ways. For modeling ways Local_1 (based on NUTS 1), Local_2 (based on static environmental features), and Local_3 (based on time series of infection cases), we train 16, 5, and 2 local models, respectively.

All three groups of local models are trained based on the optimized parameters. Similar to Table 4, Table 7 displays the average value of four indicators in 400 regions. The Local_2 can achieve the best result in all four indicators (MAE is 26.118, MSE is 2771.404, RMSE is 31.185, and Cosine is 0.761), which shows that the spatial partition based on environmental attributes can better reflect the spatial heterogeneity of infection patterns than other two. The results indicate that the choice of spatial partitions of local modeling is crucial to the model performance and should be designed according to the actual problem.

**Table 7:** Comparison of forecasting performance between three local modeling ways.

Modeling	MAE	MSE	RMSE	Cosine
Local_1	27.291	2953.607	32.430	0.745
Local_2	**26.118**	**2771.404**	**31.185**	**0.761**
Local_3	26.291	2789.320	31.370	0.741

Better results are in bold.

## Methods

### Global- and local modeling for XGBoost on simulated data

Spatial heterogeneity^5^ will lead to the fact that in geospatial studies, changes in spatial location may cause changes in the equation or the parameters of the relationship between the input variables and the target variable. Besides, the data characteristics for each studied region may also vary greatly. Therefore, the simulated data under three basic mathematical functions (i.e., Linear function, Power function, and Trigonometric function) are generated to investigate the performance of the XGBoost algorithm under global- and local modeling ways. In addition, we also study the impact of sample size and value range on the model performance.

It is usually assumed that the phenomenon investigated is random and follows Normal distribution for statistical research. However, existing studies have proven that the geographical system is complex and exhibits non-Normal distributions (e.g., Exponential distribution and Power law distribution) for geographical phenomena, such as urban population density^29,30^, city sizes^31^, street network connectivity^32^, and the frequency of toponyms in Points of Interest (POIs)^33^. Therefore, to ensure that the distribution of generated data is consistent with the real data in geospatial studies, we resample the generated data under two distributions (i.e., Normal distribution and Pareto distribution) before modeling training.

Given *X* as the model input and *Y* as the model output, $W_1$, $W_2$, and $W_3$ are three weights for *X*, $B_1$, $B_2$, and $B_3$ are random numbers as noises, and Eqs. (1), (2), and (3) are three relationships between independent and dependent variables under the linear function, the power function, and the trigonometric function, respectively. *X* is a random number generated according to the corresponding value range, and *Y* is generated based on the corresponding equation and generated *X* and *B*.

1 $$\begin{aligned} & Y=W_1X+B_1, \end{aligned}$$

2 $$\begin{aligned} & Y=W_2X^2+B_2 \end{aligned}$$

3 $$\begin{aligned} & Y=W_3(\sin (X)+1)+B_3 \end{aligned}$$

For each generation step, we select two of the three equations and independently generate two sets of data under different relationships between variables *X* and *Y*. To make the comparison of the global- and local modeling ways rigorous, we only change one or a group of parameters for each aspect while keeping other experimental parameters unchanged. Generally, the data under each equation have a sample size of 1000, which is resampled from 10,000 original generated data according to the probability density of Normal distribution. $B_1$, $B_2$, and $B_3$ are independently generated random numbers between 0 and 100. $W_1=5$, $W_2=10$, and $W_3=500$. The value ranges of *X* under Eqs. (1), (2), and (3) are between 0–200, 0–10, and 0–2$\pi$, respectively. This ensures that the value range of generated Y based on different equations is the same, i.e., 0–1000 without the noise *B*.

The train data and test data of XGBoost are independently generated twice with the same parameters. This ensures that the test data have the same distribution characteristics as the train data and the model does not learn the test samples. The global modeling of XGBoost is to train one model based on the combination of two sets of data with a distinguishing number (e.g., ($x_1$, 1),$y_1$, and ($x_2$,2),$y_2$. $x_1,x_2$ denote two random *X* and $y_1,y_2$ denote two generated *Y* under the corresponding Eqs. (1) and (2)). This makes the global- and local modeling ways to learn the same information, allowing for a fair comparison. The local modeling trains two individual models based on the corresponding relationship of generated data. The XGBoost algorithm is implemented based on the Python package XGBoost (https://xgboost.readthedocs.io/en/stable/). For each model training, all the parameters are defaults avoiding human intervention except for the different train data.

### Global- and local modeling for XGBoost on German COVID-19 infection forecasting

COVID-19 has spread globally since 2020, and many researchers have utilized AI methods to predict this epidemic’s time-series characteristics (e.g., daily new cases)^34–36^. Since countries and regions worldwide have different epidemic-against policies, economic levels, and medical levels, spatial heterogeneity should also be considered in COVID-19 infection forecasting. However, except for studies specifically focusing on the spread of the epidemic, research that simply applies AI models to epidemic-related tasks less considers the differences between global- and local modeling approaches. Only a few studies provide the modeling details of AI methods (e.g., Kumar et al.^37^ trained models for different countries separately), while the modeling approaches of other studies have less clear descriptions^38,39^. It should be noted that the purpose of this experiment is to compare the global- and local modeling approaches of the XGBoost algorithm in geospatial studies by taking German COVID-19 infection forecasting as an example. Therefore, more complex and accurate infectious disease models such as the Susceptible-Infected-Recovered (SIR) model^40,41^ are not discussed in this study.

#### Study area and data

The study area of this research is Germany. Considering the available data and the number of regions for comparing the two modeling ways, i.e., local- and global, we select the county level (i.e., NUTS 3) as the spatial resolution. There are 400 regions in the NUTS 3 level in Germany. The models are trained to forecast the daily new COVID-19 cases in each region, and the raw infection data in Germany are provided to the public by the Robert Koch Institute (RKI) (https://github.com/robert-koch-institut/SARS-CoV-2-Infektionen_in_Deutschland). In addition to historical COVID-19 infection data, we also collect multiple data recording information from several perspectives as train data, including epidemic-related data [policy index from a platform Infas 360 (https://www.healthcare-datenplattform.de/en/)], privacy-protected human activity data [i.e., contacts^42^ and traffic volumes (from the HERE company)], and environmental data [temperature and humidity from Deutscher Wetterdienst (DWD) (https://opendata.dwd.de/climate_environment/CDC/observations_germany/climate/)]. These data collected in the same spatio-temporal range record human activity characteristics and environmental characteristics, providing more information for the forecasting of COVID-19 infection.

Multiple raw data are organized into time series to reflect the daily variation of specific variables. The raw climate data from DWD are based on the observation stations. To get the daily temperature and humidity of each county, we use the inverse distance weighted algorithm to perform spatial interpolation based on all the stations in Germany. The spatial statistical units of the original traffic data are the street segments, and we also aggregate the data at the county level. In terms of policy and contact data, only the state level (i.e., NUTS 1) is available. To ensure that the formats of organized multiple data are consistent, we assign the variable value at the state level to all counties contained in this region. Although it brings additional inaccuracies, e.g., the epidemic-related policy indexes in different counties within the same state are not entirely the same, it actually provides more information for the COVID-19 infection forecasting task. The available periods for multiple time series are from 1 March 2020 to 31 December 2021, almost covering the COVID-19 outbreak. Table 8 provides examples of processed data.

**Table 8:** Example of processed data.

Date	NUTS3	Cases	Humidity	Temperature	Traffic	Contact	Policy
2020-03-01	DE111	2	71.523	6.907	1,669,325	13.766	0.000
2021-12-24	DEG0J	87	89.165	4.673	1,023,953	9.846	44.449
2021-12-25	DE115	44	91.835	1.830	1,352,827	10.459	41.226

#### Local- and global modeling

Table 8 shows that the variables have largely different value ranges (e.g., traffic volume and policy index). To eliminate the differences, each time series is processed by Z-Score normalization. We divide the organized multiple time series into train data and test data according to the date, i.e., train data are from 1 March 2020 to 24 December 2021, and test data are from 25 December 2021 to the end of 2021. In this study, we utilize the XGBoost algorithm^18^ to forecast COVID-19 infection cases in Germany. For each forecasting step, the historical data of the past four weeks (i.e., 28 days) are used as a model input to forecast the infection cases in the next week (i.e., 7 days). According to such formats, i.e., 35 days as segments, the entire time series is split by the corresponding length (e.g., from 1 March 2020 to 4 April 2020, and from 2 March 2020 to 5 April 2020) for the convenience of model training. The experimental time series data of each NUTS 3 region have no missing values. After data organization, the sample size of train data in each NUTS 3 region is equal to 629.

For the comparison of global- and local modeling of XGBoost, we train several models separately. In terms of global modeling, only one model is trained based on data from 400 NUTS 3 regions in Germany. The sample size of train data is therefore $629 \times 400 = 251{,}600$. For the local modeling, 400 regions are grouped into several partitions, and we independently train the specific local model for each partition based on the corresponding data. The train data of the local model do not include information outside the specific partition. For this study, we select the spatial partitions based on NUTS 1 (i.e., state level) as an example for local modeling, because these NUTS 1 regions have characteristic differences (e.g., population density, area, and epidemic prevention policy) in COVID-19 infection across regions, and those characteristics can be assumed to be consistent within the regions. Besides, these administrative boundaries can be directly determined and no further spatial processing is required. The number of local models that need to be trained based on NUTS 1 partitions is 16. Since each NUTS 1 region contains a different number of NUTS 3 regions, the sample size of train data for each local model is also different (Table 9). To fairly compare global- and local modeling, the NUTS ID that identified the specific region is also added as an input variable for global modeling to make the data utilized in the two modeling ways consistent^43^.

**Table 9:** Hyperparameter and train data information of global and local models.

Modeling		Estimators	Max depth	Learning rate	Train size	Max daily infection
Global		29	4	0.395	251,600	2809
Local	1	15	4	0.247	27,676	806
	2	25	6	0.145	60,384	2220
	3	20	2	0.346	629	2809
	4	37	6	0.261	11,322	518
	5	40	1	0.258	1258	409
	6	36	5	0.167	629	1225
	7	34	5	0.419	16,354	553
	8	35	3	0.065	5032	556
	9	17	3	0.458	28,305	687
	10	30	4	0.401	33,337	1258
	11	37	6	0.261	22,644	234
	12	23	4	0.363	3774	332
	13	26	4	0.217	8177	1589
	14	19	3	0.183	8806	948
	15	47	3	0.156	9435	136
	16	20	2	0.346	13,838	744

In this study, the XGBoost algorithm^18^ to forecast COVID-19 infection is implemented based on the same Python package as the simulation experiments and also the MultiOutputRegressor function of the scikit-learn library^28^ (https://scikit-learn.org/stable/modules/generated/sklearn.multioutput.MultiOutputRegressor.html). To fairly compare the local- and global modeling of XGBoost, we utilize the Bayesian Optimization^44^ (https://github.com/bayesian-optimization/BayesianOptimization) method to tune three parameters (i.e., the number of estimators, the max depth of trees, and the learning rate) of each model to ensure that both global and local models can be fully trained and achieve the best results. Besides, we perform a 5-fold cross-validation on the train data to ensure that the optimized parameters are robust. The value ranges of the number of estimators, the max depth, and the learning rate are 1 to 50, 1 to 10, and 0 to 1, respectively. The optimized parameters used for global and local XGBoost models and the information of corresponding train data are displayed in Table 9.

## Conclusions

This study compares the global- and local modeling ways of XGBoost in geospatial analysis tasks. For the experiments based on simulated data, we study the impacts of data distribution, data size, and value range on model performance and find that when the data under different relationships are balanced and the value ranges are similar, global modeling of XGBoost is better in most cases; otherwise, local modeling is stable and better, especially for the secondary data. For the task of German COVID-19 infection forecasting, the results of the two modeling ways of XGBoost have no significant difference in the temporal and spatial dimensions. The spatial partition of the study area requires extra attention for local modeling and will affect the model performance. Besides, the sub-models of local modeling have the potential to be trained by parallel computing due to the independent training process.

There are still some points in this study that can be further improved and studied. In terms of the comparison between global- and local modeling ways of XGBoost, the conclusions drawn in this study are based only on existing simulation experiments and a case study of German COVID-19 infection forecasting, it is worth conducting more experiments under complex relationships between *X* and *Y*, or applying to other cases. In terms of the COVID-19 infection forecasting task, this study focuses more on comparing the two modeling ways of XGBoost, so the experiments are designed for fair comparison (e.g., controlling train data and model parameters). It is relatively simple to finish the epidemic forecasting task. Besides, some interesting aspects (e.g., privacy protection) of the comparison between global- and local modeling ways of XGBoost can also be explored in future work.

As mentioned in the introduction, although AI techniques have many applications in geospatial analysis, few studies have the direct purpose of comparing the global- and local modeling ways of a particular algorithm (e.g., XGBoost). The conclusion of this research is consistent with some previous studies, i.e., both global- and local modeling ways of XGBoost have their advantages and the modeling approach selection needs to follow the actual cases in geospatial studies, e.g., data volume and quality, time-consuming requirements, and preliminary results of the trained model. In terms of the direction of AI applications with the consideration of spatial heterogeneity, Xie et al.^45^ designed new methods for spatial-heterogeneity-based adaptive learning to train deep neural networks, which can automatically consider the spatial heterogeneity of research data. Cheng et al.^46^ proposed a concept *decomposition learning*, which combined the global- and local modeling ways. Ideally, global modeling can solve most general problems when the spatial heterogeneity of studied data is not significant, while local modeling can supplement it for specific cases.

## Data Availability

The human activity data analyzed during the current study including contacts and traffic volumes are not publicly available and only for internal project members. Other datasets used and analyzed during the current study are available from the corresponding author on reasonable request.
